# Cardiovascular Disease in Testicular Cancer Survivors: Identification of Risk Factors and Impact on Quality of Life

**DOI:** 10.1200/JCO.22.01016

**Published:** 2023-04-18

**Authors:** Sjoukje Lubberts, Harmke J. Groot, Ronald de Wit, Sasja Mulder, Johannes A. Witjes, J. Martijn Kerst, Gerard Groenewegen, Joop D. Lefrandt, Flora E. van Leeuwen, Janine Nuver, Michael Schaapveld, Jourik A. Gietema

**Affiliations:** ^1^ Department of Medical Oncology, University Medical Center Groningen, University of Groningen, Groningen, the Netherlands; ^2^ Department of Epidemiology, Netherlands Cancer Institute, Amsterdam, the Netherlands; ^3^ Department of Medical Oncology, Erasmus Medical Center, Rotterdam, the Netherlands; ^4^ Department of Medical Oncology, Radboud University Medical Center, Nijmegen, the Netherlands; ^5^ Department of Urology, Radboud University Medical Center, Nijmegen, the Netherlands; ^6^ Department of Medical Oncology, Netherlands Cancer Institute, Amsterdam, the Netherlands; ^7^ Department of Medical Oncology, University Medical Center Utrecht, Utrecht, the Netherlands; ^8^ Department of Vascular Medicine, University Medical Center Groningen, University of Groningen, Groningen, the Netherlands

## Abstract

Testicular cancer (TC) treatment is clearly associated with cardiovascular morbidity and mortality. To enable development of preventive strategies for cardiovascular disease (CVD), we assessed cardiometabolic risk factors and quality of life (QoL) in TC survivors.

**METHODS:**

Incidence of coronary artery disease, myocardial infarction, and heart failure after TC treatment was assessed in a multicenter cohort comprising 4,748 patients treated at the age of 12-50 years between 1976 and 2007. Patients who had developed CVD and a random sample from the cohort (subcohort) received a questionnaire on cardiometabolic risk factors and QoL. A subgroup of responders in the subcohort additionally underwent clinical evaluation of cardiovascular risk factors.

**RESULTS:**

After a median follow-up of 16 years, 272 patients had developed CVD. Compared with orchidectomy only, cisplatin combination chemotherapy was associated with an increased CVD risk (hazard ratio [HR], 1.9; 95% CI, 1.1 to 3.1). Patients who were obese or a smoker at diagnosis (HR, 4.6; 95% CI, 2.0 to 10.0 and HR, 1.7; 95% CI, 1.1 to 2.4, respectively), developed Raynaud's phenomenon (HR, 1.9; 95% CI, 1.1 to 3.6) or dyslipidemia (HR, 2.8; 95% CI, 1.6 to 4.7) or had a positive family history for CVD (HR, 2.9; 95% CI, 1.7 to 4.9) had higher CVD risk. More TC survivors with CVD reported inferior QoL on physical domains than survivors who did not develop CVD. Of 304 TC survivors who underwent clinical evaluation for cardiovascular risk factors (median age at assessment: 51 years), 86% had dyslipidemia, 50% had hypertension, and 35% had metabolic syndrome, irrespective of treatment.

**CONCLUSION:**

Cardiovascular events in TC survivors impair QoL. Many TC survivors have undetected cardiovascular risk factors. We advocate early lifestyle adjustments and lifelong follow-up with low-threshold treatment of cardiovascular risk factors, especially in obese and smoking patients treated with platinum-based chemotherapy.

## INTRODUCTION

Since the introduction of platinum-based chemotherapy, survival of patients with testicular cancer (TC) has improved substantially. Five-year survival rates now range between 99% for localized and 70% for poor risk disseminated disease.^1,2^ In addition, the incidence of TC is increasing. As a result, the number of TC survivors is growing rapidly.^3^ Therefore, prevention or early detection of late adverse effects of TC treatment has become increasingly important. Previous research has shown that TC treatment, particularly platinum-based chemotherapy, is associated with increased risk of cardiovascular morbidity and mortality, probably resulting from vascular damage.^4-7^ The mechanisms behind vascular damage after TC treatment are not yet fully understood but may involve development of endothelial dysfunction. This association has been shown in vitro and in vivo.^8-11^ Endothelial dysfunction is influenced by cardiovascular risk factors, and TC treatment has been shown to be associated with development of an unfavorable cardiovascular risk profile, including dyslipidemia, hypertension, insulin resistance, and overweight. The latter are all aspects of the metabolic syndrome, which may affect up to 25% of TC survivors treated with platinum-based chemotherapy.^5,12-16^

**CONTEXT**

**Key Objective**
How can we identify which patients with testicular cancer (TC) are at risk to develop cardiovascular disease (CVD) after treatment?
**Knowledge Generated**
Knowledge of CVD development after TC is relevant because CVD leads to decreased quality of life. Platinum-based chemotherapy, obesity, and smoking at diagnosis lead to increased CVD risk. Patients who developed Raynaud's phenomena after treatment or dyslipidemia during follow-up were at increased risk for CVD development.
**Relevance *(M.A. Carducci)***
This manuscript reinforces the long term survivorship risks for TC survivors, many diagnosed as adolescent and young adults. Platinum-based chemotherapy increases the long term risk of vascular disease. Lifelong preventive care is warranted to reduce risk of CVD through known mitigation efforts.*
  *Relevance section written by *JCO* Associate Editor Michael A. Carducci, MD.

In this study, we evaluated risk factors for development of cardiovascular disease (CVD) after TC treatment. We assessed cardiometabolic risk factors at diagnosis and during follow-up, along with well-known adverse treatment effects such as hypogonadism and Raynaud's phenomenon. Our secondary objectives were to investigate the impact of CVD on quality of life (QoL) and determine the actual presence of cardiometabolic risk factors in a random sample of TC survivors. Previous major studies looked mostly at treatment associations with CVD risk and did not assess in depth the characteristics of TC survivors who developed CVD.^4-7,17^ Our study is the first to investigate a possible association between early adverse treatment effects such as Raynaud's phenomena and subsequent CVD development.

## METHODS

### Study Design

Five large Dutch TC treatment centers participated in this study: University Medical Center Groningen (UMCG), Netherlands Cancer Institute/Antoni van Leeuwenhoek Hospital Amsterdam (NKI/AVL), Erasmus Medical Center Rotterdam (EMC), Radboud University Medical Center Nijmegen (RUMC), and University Medical Center Utrecht (UMCU; ClinicalTrials.gov identifier: NCT02276430). These hospitals provided access to the medical records of 4,748 patients with TC who had completed their treatments between 1976 and 2007. Their age at TC diagnosis ranged from 12 years to 50 years. Patients who developed CVD after TC were identified in regular oncological follow-up or through contact with their general practitioner, as reported previously.^18^ CVD was defined as myocardial infarction, proven coronary artery disease (Common Terminology Criteria for Adverse Events version 4 [CTCAE-4]: grade 2 or higher, ICD10 I20-25), or congestive heart failure (CTCAE-4 grade 2 or higher, ICD-10 I50). CVD before TC diagnosis was considered an exclusion criterion for analysis as a CVD case. A case-cohort design was chosen (Data Supplement [Box 1], online only) to reduce the number of medical records to be abstracted while maintaining statistical power.^19^ Patients who developed CVD were compared with a hospital-stratified subcohort, comprising 15% of the cohort in the EMC, RUMC, and UMCU and 25% of the cohort in the coordinating hospitals NKI/AVL and UMCG, which was randomly selected from the base cohort. It consisted of 925 patients, of whom 54 developed CVD. We abstracted treatment data from the medical records for all patients in the base cohort who developed CVD (cases) and all patients in the hospital-stratified subcohort (from now on: subcohort patients). The medical records specified their primary TC treatment and any relapse or contralateral TC treatment .

All patients with TC underwent hemiorchidectomy. For early-stage seminoma, orchidectomy was usually followed by radiotherapy, mostly targeting infradiaphragmatic para-aortic, ipsilateral iliac, and inguinal lymph nodes, with doses ranging from 30 to 35 Gy.^20^ From the mid-1980s, radiation doses gradually decreased to 26 Gy. Patients with stage II-IV seminoma and nonseminoma were most frequently treated with cisplatin-containing chemotherapy—initially with cisplatin, vinblastine, and bleomycin and since the mid-1980s with bleomycin, etoposide, and cisplatin.^21^

**FIG 1.**
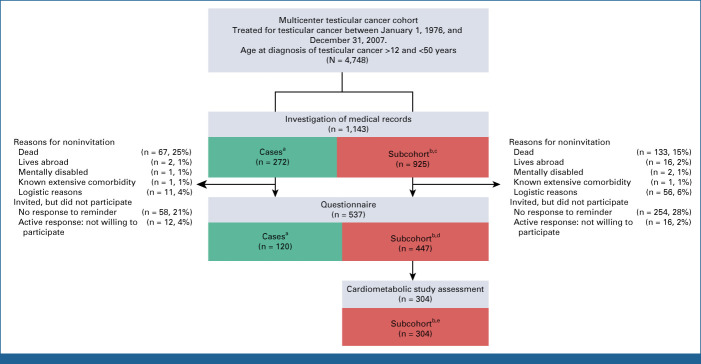
The numbers of enrolled patients in this study, including reasons for noninvitation or nonparticipation. ^a^Cases: patients who developed cardiovascular disease. ^b^Subcohort: patients in the random sample from the multicenter testicular cancer cohort. ^c^Of whom 54 patients developed cardiovascular disease. ^d^Of whom 30 patients developed cardiovascular disease. ^e^Of whom 18 patients developed cardiovascular disease.

During 2015-2017, all patients in the base cohort who had developed CVD (cases) and all subcohort patients who were alive were invited to complete a risk factor questionnaire. The questionnaire assessed life style, use of medication, family history for CVD, QoL (measured by the SF-36),^22^ and known adverse treatment effects (Raynaud's phenomenon, neurotoxicity, ototoxicity, and fatigue).^23^ In total, 717 patients were invited to complete the questionnaire, including 190 with CVD. The questionnaire was completed by 120 of 190 patients with CVD (63%) and 447 of 717 subcohort patients (62%) in total. Subcohort patients who completed the questionnaire and were younger than 40 years at TC diagnosis and younger than 75 years at inclusion were then invited to participate in a cardiometabolic assessment. A total of 304 subcohort patients participated in the cardiometabolic study assessment, of whom 18 (6%) had developed CVD. An overview of study participation rates and reasons for noninvitation or nonparticipation are shown in Figure 1. The medical ethics committee (UMCG) approved this study. All participants gave written informed consent.

### Cardiometabolic Study Procedures

The cardiometabolic study assessment consisted of physical examination, including anthropometrics (weight, height, and waist/hip circumference) and blood pressure measurement. Patients could visit one of the participating hospitals or their general practitioner for the anthropometrics and blood pressure measurements while sending their fasting blood samples to a central laboratory (UMCG). Hypertension was defined as a systolic blood pressure ≥140 mmHg, a diastolic blood pressure ≥90 mmHg, or use of antihypertensive drugs.^24^ A fasting blood sample was drawn to establish lipid profile, glucose levels, and hormonal status. Dyslipidemia was defined as a fasting total cholesterol ≥5.2 mmol/L (201 mg/dL), LDL ≥2.5 mmol/L (97 mg/dL), HDL <1.04 mmol/L (40.2 mg/dL), or triglycerides >4.5 mmol/L (399 mg/dL) or usage of lipid lowering medication.^25,26^ Diabetes mellitus was defined as glucose ≥7.0 mmol/L (126 mg/dL) or use of blood glucose lowering medication and prediabetes as glucose ≥5.6 to <7.0 mmol/L (101 mg/dL).^27^ The metabolic syndrome was defined according to the National Cholesterol Education Program Adult Treatment Panel III.^28^ In the blood samples, biomarkers for endothelial function (von Willebrand factor), hemostasis (coagulation factor VIII, tissue plasminogen activator [t-PA], plasminogen activator inhibitor 1 [PAI-1], fibrinogen), and inflammation (high sensitivity C-reactive protein) were measured. This biomarker selection was based on earlier studies of our group.^9-11^ Hypogonadism was defined as serum testosterone concentration <10 nmol/L and/or a LH concentration ≥10 U/L or testosterone use. All blood samples were drawn between 7:30 and 11:30 a.m. to limit circadian variation. A morning urine sample was tested for microalbuminuria, defined as 2.5-25 mg albumin per mmol creatinine. Macroalbuminuria was defined as >25 mg albumin per mmol creatinine.

### Statistics

Associations of TC treatment and cardiovascular risk factors with CVD risk were assessed in multivariable Cox regression models and presented as hazard ratios (HRs). Time at risk started at the date of orchidectomy and ended at the date of diagnosis of the first CVD of interest for patients with CVD (myocardial infarction, coronary artery disease, or congestive heart failure [ICD-10 I20-25, I50]). Patients without CVD were censored at date of death, emigration, or last follow-up, whichever came first. Barlow's weights were used to adjust the partial likelihood function for case-cohort analysis^19,29^ (Data Supplement [Box 2]). Regression analyses were adjusted for age at TC diagnosis. Cardiovascular risk factors which occurred during follow-up were modeled as time varying covariates. Missing baseline data on cumulative treatment and cardiovascular risk factors at diagnosis were handled by performing multiple imputation by chained equations (Data Supplement [Box 2]). Missing information from the questionnaires was handled by introducing a dummy variable in the model. Competing risk analysis was performed using death due to other causes than CVD as competing event.^30^ Differences in QoL were assessed using unweighted linear regression with each scalar response to the items of the QoL questionnaire treated as a separate dependent variable, corrected for age at completion of the questionnaire, years between TC diagnosis, and completion of the questionnaire and years after CVD (which was zero for noncases). *P* values ≤.05 were considered significant. STATA statistical software (version SE13, StataCorp, College Station, TX) was used for analysis.

## RESULTS

### Patients With CVD

After a median follow-up of 16.1 years (IQR, 9.7-24.5 years; range, 0-38.5 years), 272 TC survivors in the study population of 4,748 patients had developed CVD: 64% of them had experienced a myocardial infarction and 28% had coronary artery disease without infarction (Table 1). Of these patients, 16% developed heart failure afterward. Of the CVD events, 6% were fatal (n = 16).

**TABLE 1.**
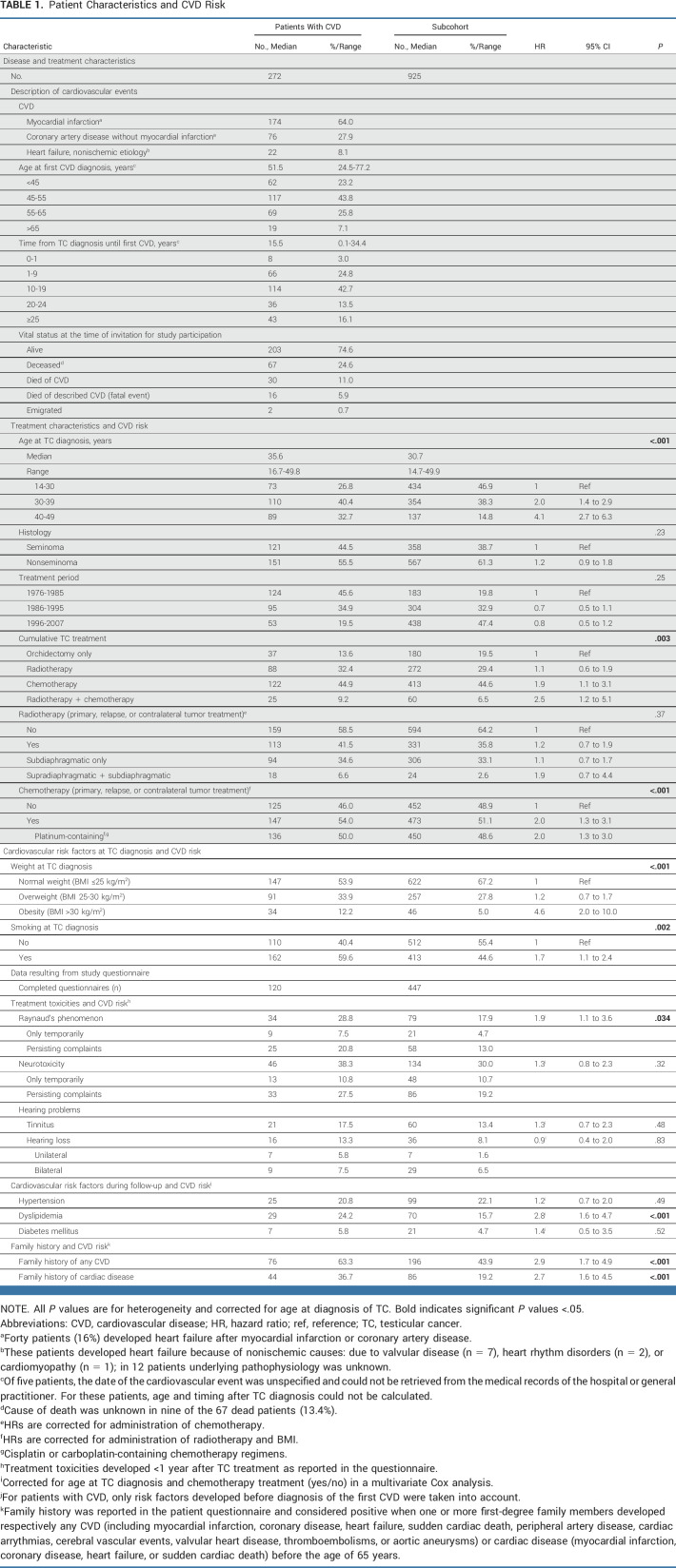
Patient Characteristics and CVD Risk

### Impact on QoL

Of the TC survivors who completed the risk factor questionnaire (n = 537; Fig 1), those who developed CVD after TC treatment reported a lower QoL on several domains than TC survivors without CVD (Table 2). TC survivors with CVD reported lower physical functioning (median score, 80 [IQR, 55-95] compared with 95 [IQR, 85-100] in subcohort patients without CVD, *P* < .001), which was accompanied by role limitations because of physical health. TC survivors with CVD also reported less energy and vitality, experienced more bodily pain (*P* = .002 and *P* = .036, respectively), and had a lower general health score than the TC survivors without CVD (*P* < .001). Furthermore, TC survivors who developed CVD reported more fatigue than patients who did not (*P* < .003). Social and mental health was not impaired in TC survivors with CVD.

**TABLE 2.**
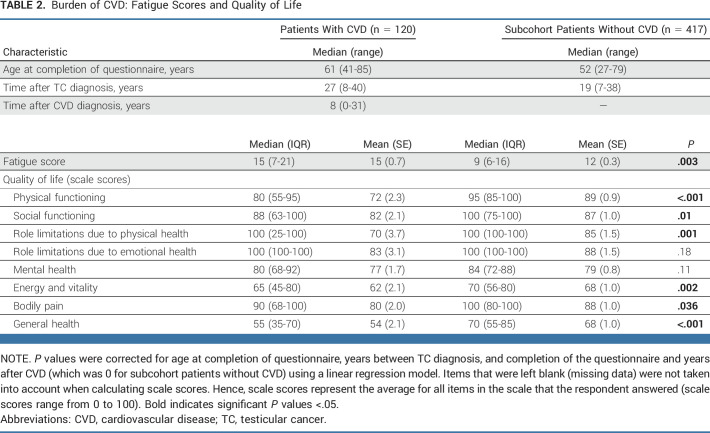
Burden of CVD: Fatigue Scores and Quality of Life

### Risk Factors Associated With CVD Development

Compared with orchidectomy only, treatment with chemotherapy was associated with increased CVD risk (HR, 1.9; 95% CI, 1.1 to 3.1) and when patients also received radiotherapy, this risk further increased (HR, 2.5; 95% CI, 1.2 to 5.1). Treatment with radiotherapy only was not associated with CVD risk (HR, 1.1; 95% CI, 0.6 to 1.9; Table 1).

TC survivors with CVD were older at TC diagnosis than subcohort patients (median age, 35.6 *v* 30.7 years; *P* < .0001; Table 1). Presence of obesity at TC diagnosis (HR, 4.6; 95% CI, 2.0 to 10.0) and smoking at TC diagnosis (HR, 1.7; 95% CI, 1.1 to 2.4) was associated with increased CVD risk (Table 1).

Analysis of raw data (without imputation of missing variables), and analysis only including patients with complete data, did not notably change the results (Data Supplement [Tables 1, 2a, and 2b]). Competing risk analysis using death due to other causes than CVD as competing event resulted in estimates pointing in the same direction (Data Supplement [Table 3]). There were no significant differences in baseline characteristics between patients participating in the study questionnaire and patients who did not participate (Data Supplement [Table 4]).

TC survivors who developed dyslipidemia during follow-up had an increased CVD risk (HR, 2.8; 95% CI, 1.6 to 4.7). Patients who reported a positive family history for CVD were more prone to develop CVD (HR, 2.9; 95% CI, 1.7 to 4.9). Patients who had developed Raynaud's phenomenon after TC treatment seemed to have a higher risk of developing CVD (HR, 1.9; 95% CI, 1.1 to 3.6), correcting for administration of chemotherapy (Table 1).

### Study Visit for Cardiometabolic Assessment

The cardiometabolic assessment (304 patients participating) took place at a median follow-up duration of 22 years after TC diagnosis (range, 8-39 years) and a median age of 51 years (range, 27-74 years; Table 3). Participants often had hypertension (50%), of which 48% was untreated. The prevalence of dyslipidemia was 86%, of which 82% was untreated. Dyslipidemia mostly presented as high LDL (82%) and/or high total cholesterol (55%). Prediabetes was also prevalent: 40% of the participants had a high fasting glucose. Of the participating TC survivors, 35% met the criteria for metabolic syndrome. Hypogonadism was present in 34%. Patients treated with orchidectomy only rarely reported Raynaud's phenomenon (Table 4).

**TABLE 3.**
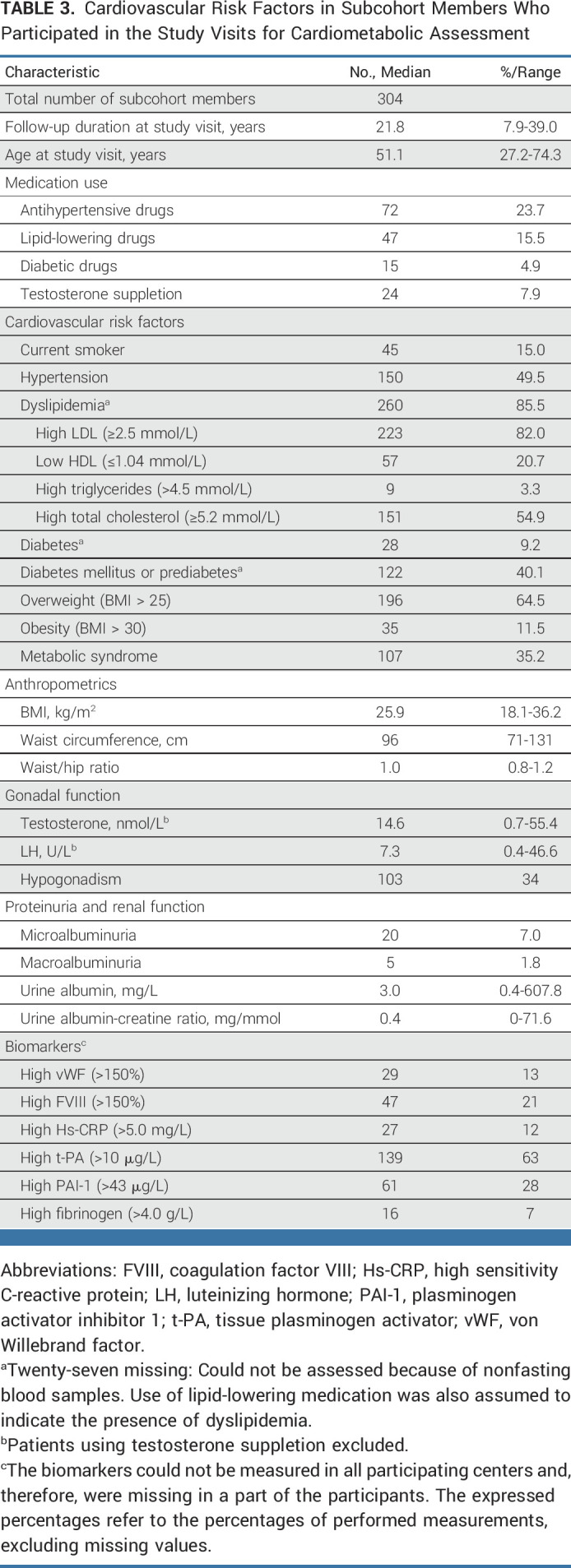
Cardiovascular Risk Factors in Subcohort Members Who Participated in the Study Visits for Cardiometabolic Assessment

**TABLE 4.**
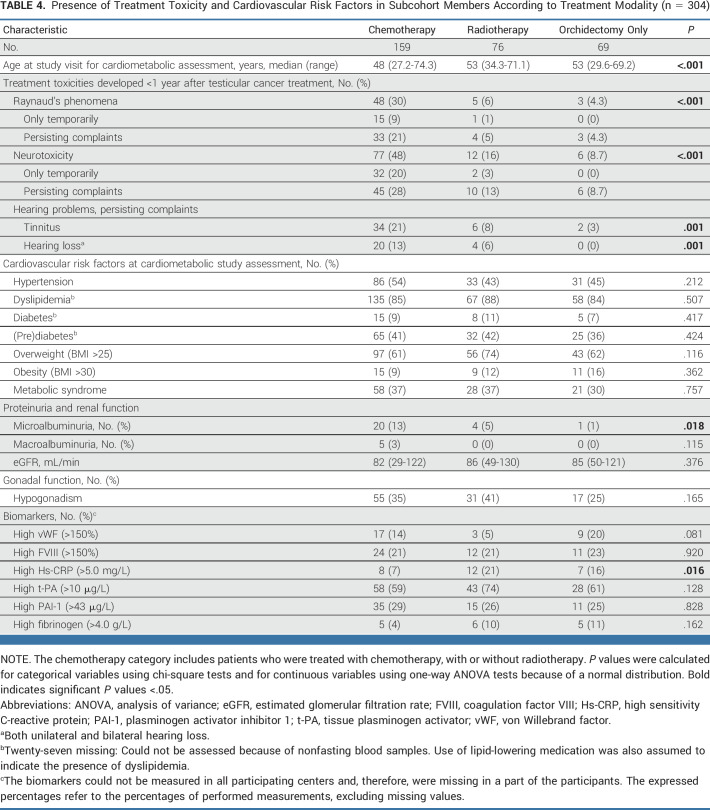
Presence of Treatment Toxicity and Cardiovascular Risk Factors in Subcohort Members According to Treatment Modality (n = 304)

Most cardiovascular risk factors were equally present in patients treated with orchidectomy only and in patients treated with chemotherapy or radiotherapy; only microalbuminuria was more frequently present in patients treated with chemotherapy (Table 4).

Prevalence of cardiovascular risk factors generally increased with increasing age at the time of the study visit (Data Supplement [Table 5]). However, the prevalence of dyslipidemia was already 82% in patients younger than 40 years, which was similar to the prevalence among the oldest subcohort members (81% in patients older than 60 years).

At the study visit, biomarkers for endothelial dysfunction, low-grade inflammation, and hemostasis were more often elevated (Table 3), especially t-PA and PAI-1 (63% and 28% respectively). Compared with survivors who had not developed metabolic syndrome, more TC survivors with metabolic syndrome had high t-PA (81% *v* 51%; *P* < .001), high PAI-1 (44% *v* 17%; *P* < .001), and high fibrinogen (18% *v* 3%; *P* = .005).

## DISCUSSION

In this large case-cohort study with long-term follow-up, we studied risk factors contributing to the development of serious cardiovascular morbidity in TC survivors. Besides having been treated with platinum-based chemotherapy, obesity and smoking at the start of treatment, development of Raynaud's phenomenon or dyslipidemia after treatment, and a family history of CVD were important risk factors. We also studied the QoL of TC survivors. Although previous studies in unselected TC survivors showed similar health-related QoL compared with the general population,^31^ our study showed that the development of CVD after TC treatment clearly affects the survivors' QoL: patients who experienced CVD reported inferior QoL on physical domains compared with TC survivors who did not develop CVD.

In patients with TC who participated in the cardiometabolic study assessment, we observed a high prevalence of cardiovascular risk factors, which were often not previously identified and were thus untreated. This confirms the finding of a recent study that showed a higher prevalence of hypertension and hypercholesterolemia in TC survivors compared with the general male population, even from the start of treatment.^17^

Of note, cardiovascular risk factors (except for microalbuminuria) were equally prevalent in patients treated with chemotherapy, radiotherapy, or orchidectomy only. Patients with TC in our study seemed to develop cardiovascular risk factors irrespective of treatment modality, which suggests that platinum-based chemotherapy has limited influence on the development of these risk factors. The development of cardiovascular risk factors could also not be explained by factors such as the presence of hypogonadism. In addition, patients with TC appear to have an unfavorable cardiovascular risk profile even at the time of diagnosis.^11,16,17^ In our study, being obese at TC diagnosis was an important risk factor for developing CVD after treatment (HR, 4.7; 95% CI, 2.4 to 9.3). Although obesity is a known independent cardiovascular risk factor in the general population, fat tissue may also act as a body reservoir for platinum, and obesity at TC diagnosis could thus be related to circulating platinum levels long after chemotherapy.

In our study, treatment with platinum-based chemotherapy almost doubled the risk of CVD development compared with orchidectomy only (HR, 1.9; 95% CI, 1.1 to 3.1). Previous studies showed that TC treatment with chemotherapy is associated with an approximately three-fold increased risk of CVD compared with TC survivors treated with orchidectomy only.^5,18^ The exact role of bleomycin versus platinum could not be investigated in this study as both were mostly combined. From in vitro data, it is known that both agents induce alterations in endothelial cell function.^9^ Circulating platinum levels remain detectable up to 20 years after chemotherapy.^32-36^ Circulating platinum residuals may result in chronic endothelial activation and have been associated with known late effects such as hypertension.^32,35^ Whether the amount of long-term circulating platinum is also associated with CVD events has not yet been investigated. Impaired renal function is an important determinant of long-term exposure to circulating platinum residuals and may cause higher platinum levels during a longer period of time, thus contributing to endothelial activation leading to CVD.^32^ Development of Raynaud's phenomenon after treatment—which is a marker of compromised endothelial function^37^—was associated with a higher CVD risk. Another marker for endothelial activation is albuminuria, which was more prevalent in TC survivors treated with chemotherapy. Albuminuria coincides with higher vascular stiffness in TC survivors.^38^ In our study, TC survivors with the metabolic syndrome had elevated t-PA, PAI-1, and fibrinogen levels than survivors without the metabolic syndrome. This indicates an active fibrinolytic system, which also contributes to atherosclerosis.^39-41^

We, therefore, suggest a future study to determine whether a combination panel, including markers of subclinical endothelial damage (presence of Raynaud's phenomenon, albuminuria, and fibrinolytic markers), could predict which patients are at increased cardiovascular risk.

Treatment with radiotherapy only was not associated with increased CVD risk. A previous study in TC survivors^18^ and our current study suggest that supradiaphragmatic radiotherapy is associated with CVD, although on the basis of only 18 patients who received supradiaphragmatic radiotherapy.

Our study had several limitations that should be mentioned here. First, we made a cross-sectional assessment of cardiovascular risk factors, but the cardiovascular risk factors in patients who had developed CVD were derived only from questionnaires sent to the patients and their general practitioners. As a result, the information tended to be incomplete. For example, information on cardiovascular risk factors at TC diagnosis was often not mentioned in the patients' medical records (ie, information on BMI or presence of obesity was missing for 22% of the patients with CVD and for 26% of all subcohort patients). Second, some selection bias could have been present in our data as patients who experienced a higher disease burden could have been more inclined to participate in a study evaluating late adverse treatment effects. Because patients who were alive were invited for participation to the questionnaire, this resulted in selection bias on the basis of survival and age. Furthermore, discrepancies in cutoff values of cardiovascular risk factors exist between different countries.

An important strength is that this is the first study to thoroughly compare a large group of TC survivors who developed overt CVD to a large cohort. Until now, most studies have been epidemiological: to study their CVD risk, TC patients were compared to general population controls.^4-6,17,18^ Furthermore, in our random sample of TC survivors, cardiovascular risk factors and biomarkers were measured, and most participants gave their consent for a future invitation for a follow-up study to assess the association between observed cardiovascular risk factors and development of CVD.

To confirm the effectiveness of stringent treatment of modifiable cardiovascular risk factors—such as being overweight or a smoker, having high lipid levels, hypertension or a high blood glucose level—on CVD risk in TC survivors, future prospective studies are needed. Smoking cessation might require extra attention since a recent study showed that Danish TC survivors treated with chemotherapy consume more tobacco than the general population.^42^ Also of interest are studies to assess whether platelet aggregation inhibitors or statin-based, lipid-lowering therapy could prevent atherosclerosis development and signs of early vascular aging in TC survivors. These intervention studies are urgently needed; similar to adult childhood cancer survivors,^43^ cardiovascular morbidity shortens the remaining lifespan of patients with successfully treated TC^4,7,44^ and, as shown in this study, decreases their QoL.

In conclusion, TC survivors are at risk of developing CVD if they are treated with platinum-based chemotherapy, were obese or smoking at diagnosis, develop dyslipidemia during follow-up, had a positive family history of CVD, or develop Raynaud's phenomenon. TC survivors who develop CVD reported inferior QoL on physical domains compared with survivors who did not develop CVD. Many TC survivors have a high burden of cardiometabolic risk factors. As soon as possible after diagnosis, these patients should be encouraged to adopt a healthy lifestyle: stop smoking, be physically active, and maintain healthy dietary habits. Lifelong follow-up with low-threshold treatment of cardiovascular risk factors is needed, especially in obese and smoking patients treated with platinum-based chemotherapy. As oncologists, we should be working together with other health care professionals toward improving the lifespan and QoL of these young men after we have treated them successfully for TC.
